# Musical patterns for comparative epigenomics

**DOI:** 10.1186/s13148-015-0127-8

**Published:** 2015-09-08

**Authors:** David Brocks

**Affiliations:** Division of Epigenomics and Cancer Risk Factors, German Cancer Research Center, Im Neuenheimer Feld 280, 69120 Heidelberg, Germany

**Keywords:** DNA methylation, Music, Transformation, Epigenetics, Cancer

## Abstract

Scientific data has been transformed into music in order to raise awareness in the non-scientific community. While the general public is nowadays familiar with the genetic code, there is still a lack of knowledge regarding epigenetic regulation. By making use of the binary nature of the methylome, we here describe a method that transforms methylation patterns into music. The resulting musical pieces show decent complexity and allow the audible recognition between music and underlying methylation state. This approach might therefore facilitate the recognition of complex methylation patterns and increase awareness for epigenetic regulation in the general public.

**Electronic supplementary material:**

The online version of this article (doi:10.1186/s13148-015-0127-8) contains supplementary material, which is available to authorized users.

## Introduction

**Fig. 1:**
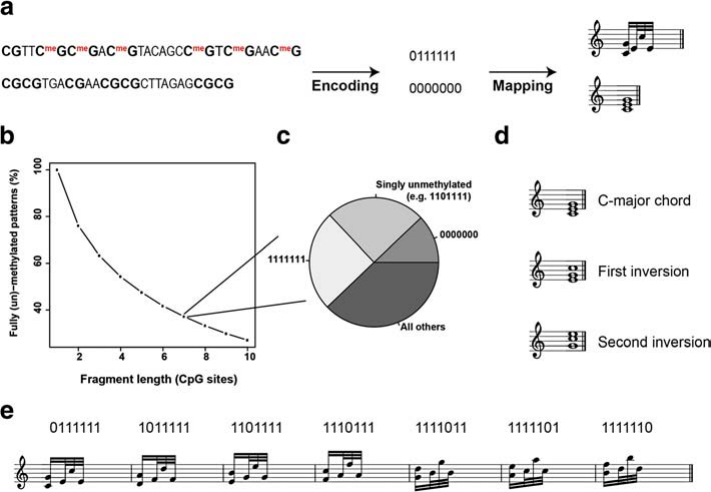
Design of a 128-note universe for transformation of methylation patterns into music. **a** Method overview: The methylation state of seven consecutive CpG sites was mapped to a note universe consisting of 128 different chords und note variations. CpG dinucleotides are *highlighted* and methyl groups are displayed by *red coloring*. **b** Percentage of clean (fully methylated or unmethylated) patterns as a function of fragment size (number of combined consecutive CpG sites). The data is based on the single embryonic stem cell methylation levels of chromosome 1. Longer fragments increase the likelihood for partially methylated patterns. **c** Methylation pattern frequency for a fragment size of length 7. Data is based on the single embryonic stem cell methylation levels of chromosome 1. Fully methylated and singly unmethylated fragments have the highest frequency. **d** First and second inversion of the C-major chord. C is the root and in the bass of the C major triad, but is not in the bass for an inverted chord. The inversions are numbered in the order their bass tones appear in the parent C major chord. **e** Note sequences assigned to the seven patterns with only one unmethylated CpG site. As the unmethylated CpG site moves from left to right, the assigned notes ascend upwards in tone. This ordering was chosen for aesthetical reasons

Modern science has grown increasingly complex and multidisciplinary, making it difficult even for active researchers to grasp theories and evidence of unrelated scientific topics. Unsurprisingly, strong skepticism or even denial may exist in the general public against robust yet complicated scientific evidence, including the theory of evolution, climate change, vaccination, or genetic engineering [1–4]. This skepticism goes along with the success of some opposing pseudoscientific areas like homeopathy, astrology, or creationism [5, 6] which, although being all heavily flawed, are apparently more engaging and easily accessible to the public. Since denial of vaccination, climate change, or genetic engineering can potentially be life-threatening, it should be the obligation of the scientific community to convey scientific data to the general public. Consequently, some interdisciplinary efforts have been made to transform complex scientific material into more appealing and accessible forms. For example, researchers working in Japan have incorporated basic concepts of developmental biology into manga-like card games [7] and another approach transformed microbial data into music by applying some principles of jazz bebop improvisation [8]. This integration of multidimensional data into audible patterns was musically appealing, but it relied on normalization of data into integers and therefore impaired the audible relationship between musical patterns and underlying experimental data. Most other approaches have focused on the conversion of a linear sequence of data into musical pieces, such as the protein amino-acid composition or the genomic DNA sequence [7, 9]. In general, these approaches also had to make a trade-off between harmony, complexity, and the audible relationship between underlying data und music. Furthermore, the genetic or amino-acid sequence can be overwhelmingly similar even between individuals of different species, making sequence comparisons musically repetitive. In contrast, the cellular methylome, i.e., the methylation status of each and every CpG dinucleotide of the genome, provides linear sequence information while being also remarkably plastic even between different developmental stages of the very same cell. Despite its importance during normal developmental as well as in common diseases like cancer, it has gained considerably less public attention than for example the genetic code. In order to facilitate public interest into epigenetic regulation, we here describe a method to transform methylome data into complex musical compositions. We make use of the fact that the methylation status of individual CpGs on a single allele is intrinsically discrete, i.e., methylated or not, and is therefore comparable to the binary code used in information technology. By fragmenting the methylome into bit strings of fixed length of 7 and mapping it to a tone universe of 128 different notes (Fig. 1a), we generate complex musical pieces while still allowing discrimination of potentially important epigenetic control regions, like long stretches of unmethylated DNA versus fully methylated or noisy fragments with intermediate methylation levels.

## Results

CpG dinucleotides are either methylated or not on a single allele, but the vast majority of methylome profiling studies report average methylation values on a continuous scale between 0 and 1 because they deal with a mixture of cells that can be highly heterogeneous in their methylation levels. In order to make use of the discrete nature of the methylome, we first focused on data obtained from single mouse embryonic stem cells [10]. Here, the combination of multiple consecutive CpG sites into a string of length *n* can provide 2^*n*^ different combinations. Consequently, the methylation state of three CpG sites would already be enough to encode for a complete monophonic octave. In reality, however, the distribution of played notes would be highly skewed towards notes encoded by fully methylated or unmethylated patterns (Fig. 1b). To decrease monotony while in parallel not exceeding a reasonable note complexity, we used the information of seven consecutive CpG sites. For this fragment size, about half of the notes played correspond to fully methylated and singly unmethylated fragments (Fig. 1c). To cover the resulting 128 different combinations, we created a note universe consisting of ten different chords with each two inversions (Fig. 1d) and four different durations (120 different chords in total). For the remaining eight combinations, we assigned note sequences consisting of a dyad followed by three monophonic notes and a 16th rest to the seven patterns with only one unmethylated CpG site (Fig. 1e) and the fully methylated fragment, respectively . The special assignments were chosen to diversify and improve the musical representation given that the highly methylated fragments have by far the highest occurrence (Fig. 1c) and, hence, disturb melody. Moreover, a singly unmethylated fragment resembles an early form of locally disordered methylation as it occurs during carcinogenesis [11]. This loss of methylation is reflected here by a noisy musical representation (dyad followed by three monophonic notes). To further facilitate the audible recognition of the methylation level of the fragments, more unmethylated patterns were generally assigned to chords with longer durations while more methylated patterns corresponded to chords with shorter durations.

**Fig. 2:**
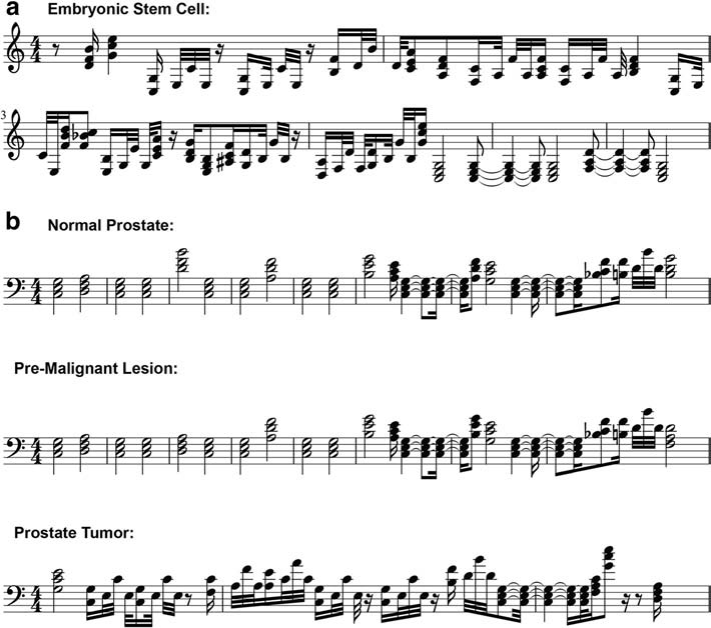
Degree of methylation divergence revealed by musical patterns. **a** Sheet music based on single ESC methylation levels of the first 238 CpG sites on chromosome 10. Transformed CpG sites range from 3,001,388 to 3,135,529 base pairs and cover no annotated gene. The sheet music sometimes ignores classical notation conventions since the musical output does not follow conventional melodic and harmonic rules. **b** Musical patterns based on *PCDHGA10* methylation levels in normal, prostatic intraepithelial neoplasia (premalignant), and prostate cancer samples. Continuous methylation was measured using Illumina 450K arrays and discretized using the following thresholds (unmethylated (0) if ≤0.5, methylated (1) if >0.5)

Figure 2a shows the sheet music based on the transformation of the mouse single ESC methylation levels of the first covered 238 CpG sites on chromosome 10. The long stretch of highly methylated DNA in the beginning is characterized by a high number of short pauses as well as the non-chord note sequences that correspond to patterns with only one unmethylated CpG site. In contrast, the unmethylated stretch of DNA at the end can be easily distinguished by the multiple consecutive C-major chords of long duration. All other chord variations with differing durations correspond to intermediate methylation levels. A longer example is shown in Additional file 1: Figure S1. Therefore, our methylome to music transformation creates complex musical pieces while simultaneously retaining the information on potentially important epigenetic control elements like long stretches of unmethylated DNA.

Next, we compared musical patterns between normal and cancerous cells in order to test the ability of our method to illustrate methylation differences between cell types. For this, we used data from Illumina 450K arrays since no single cell methylation data is currently available between healthy and malignant cells. In contrast to single cell bisulfite sequencing data, these arrays measure DNA methylation on a continuous scale (between 0 and 1) which represents the average methylation of all measured methylation states for a given CpG site within a population of cells. Furthermore, data from 450K arrays is sparse, covering less than 2 % of the more than 28 million CpG sites of the human genome. For our purpose, we therefore discretized the continuous values into either unmethylated or methylated and focused on the protocadherin gamma subfamily A 10 (*PCDHGA10*) gene that is covered more than 100 times on the array. Members of this gene family play a role in cell adhesion and signaling, and their aberrant methylation has been implicated in some human cancers [12, 13]. Figure 2b shows the sheet music based on the methylation of *PCDHGA10* in normal, premalignant, and cancerous prostate cells [14]. From the musical transformation, it becomes apparent that the unmethylated state at the 5′ start of the *PCDHGA10* gene remains conserved between normal and premalignant cells. In contrast, the prostate tumor sample shows considerable hypermethylation at this region which becomes apparent by just comparing the resulting musical pieces, showing the utility of methylation-based music to easily reveal cell-type-specific methylation differences. A comparison of multiple tumor specimens from the same patient as well as the .midi-files that correspond to all our musical transformations can be found in in Additional file 2: Figure S2 and Additional files 3, 4, 5, 6, 7 and 8, respectively.

## Discussion

The here-described methylation to music approach allows for even the untrained ear to discriminate between fragments with low, intermediate, and high levels of methylation and to judge the similarity or dissimilarity of methylomes. This framework will help to communicate the importance of epigenetic regulation as well as the vast amount of changes it undergoes during the development of human cancers to the general public. There is a particular success story where the transformation of scientific experimental data into an easily accessibly format has actually accelerated scientific progress as well as public awareness [15]. It has therefore been speculated that the natural ability of the human ear to detect subtle differences in musical patterns might facilitate the solution of biological problems [16]. An extension of the here-described methodology might help to unravel complex methylation patterns that are normally not immediately apparent. Moreover, we hope that our approach helps to arouse interest in young children for the study of natural sciences in general and epigenomic research in particular. Also, vision-impaired scientists interested in the study of methylation patterns might benefit from a musical transformation as it was the case for other fields of research [7]. In the future, we aim to incorporate further genomic and epigenomic information into our compositions, i.e., histone modifications, transcription factor binding, GC content, etc., to ideally create polyphonic musical patterns that directly allow the recognition of the chromatin state and underlying genomic context. We are also going to provide a software package that takes the users’ input to compose music based on the here-described principles. Finally, we want to stress the flexibility of our approach as it easily allows the customization of the note complexity or the emphasis on different methylation states which in the future might significantly improve the musical output.

## Material and methods

Previously reported single embryonic stem cell methylation data was downloaded from NCBI GEO (GSE56879). CpG sites without read coverage or with reads supporting both a methylated and unmethylated state (allele-specific methylation or sequencing error) were removed. The remaining 7,127,203 CpG sites of sample Ser#14 were assigned a 1 for methylated or 0 for unmethylated. Prostate tissue classification and analysis of Illumina 450K array data was performed as previously described [14]. Continuous beta values up to 0.5 and above 0.5 were discretized into 0 and 1, respectively. Binary patterns were fragmented into strings of length 7, mapped to the note universe, set to music using the open-source Java library JFugue 4.0.3, and visualized with MuseScore 1.3.

## Additional files

Additional file 1:
**Figure S1.** Sheet music based on the single embryonic stem cell methylation levels of the first 2499 CpG sites on chromosome 1. (MIDI 2 kb) Additional file 2:
**Figure S2.** Comparison of multiple tumor specimens from the same patient for the *PCDHGA10* gene. (MIDI 1 kb) Additional file 3:
**Embryonic Stem Cell Short. **(MIDI 1 kb) Additional file 4:
**Normal Prostate.** (MIDI 1 kb) Additional file 5:
**Premalignant Prostate.** (MIDI 10 kb) Additional file 6:
**Prostate Tumor.** (RAR 3 kb) Additional file 7:
**Embryonic Stem Cell Long.** (PDF 69 kb) Additional file 8:
**Different Prostate Tumors.** (TIFF 1,139 KB)
