# Supplementary figures and images for: Musical patterns for comparative epigenomics
Source: Clin Epigenetics. 2015 Sep 8;7:94. doi: 10.1186/s13148-015-0127-8 (PMC4563837; doi:10.1186/s13148-015-0127-8)

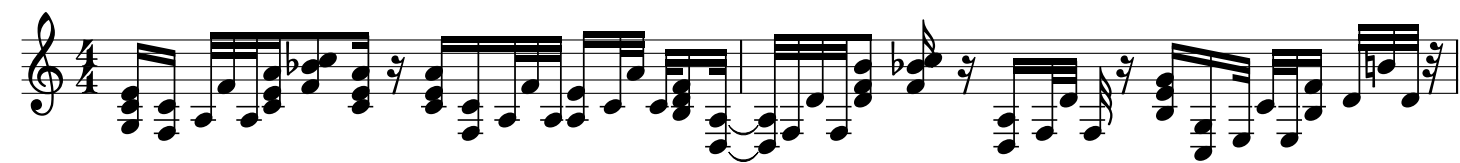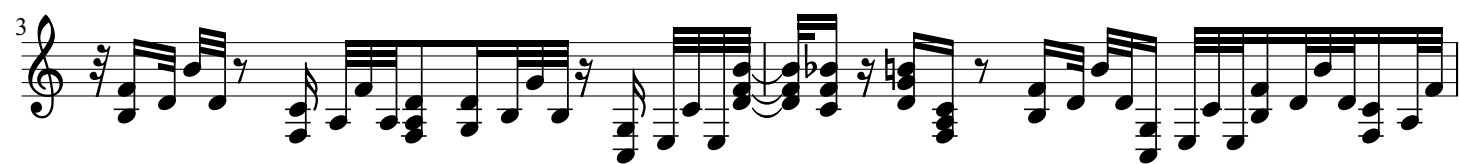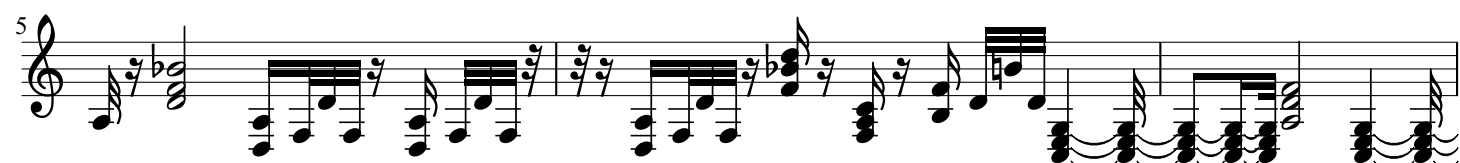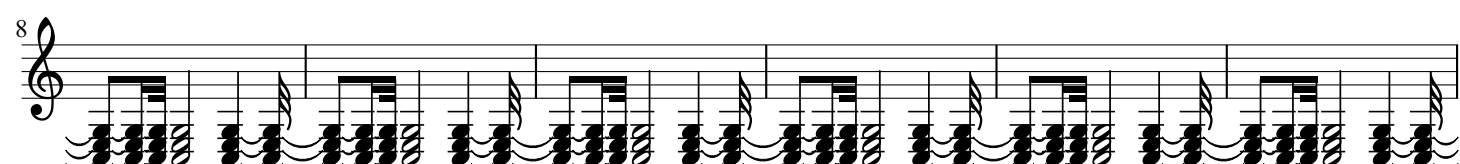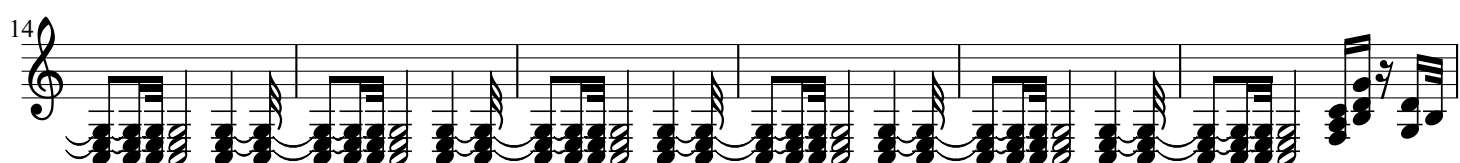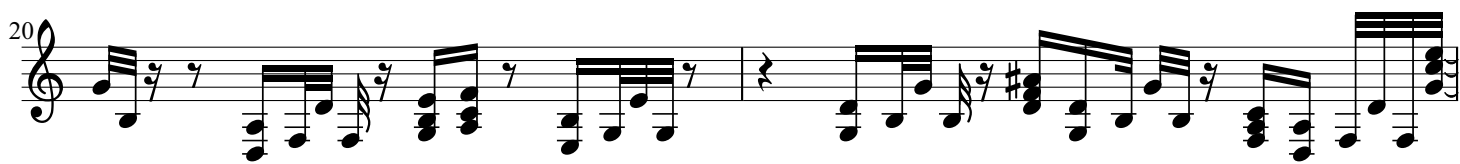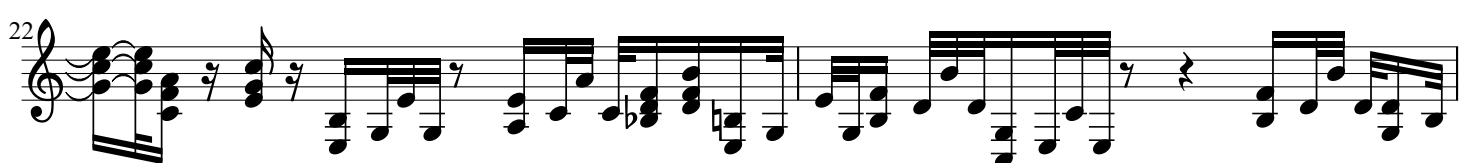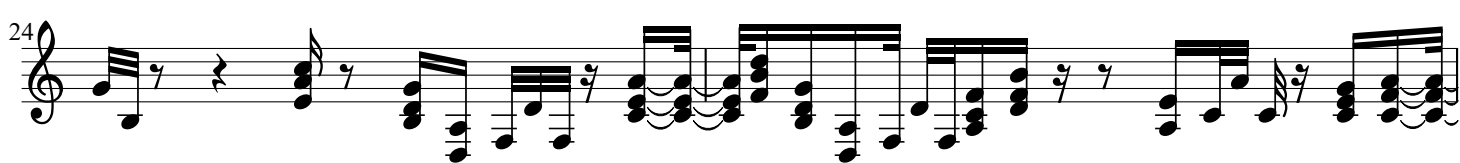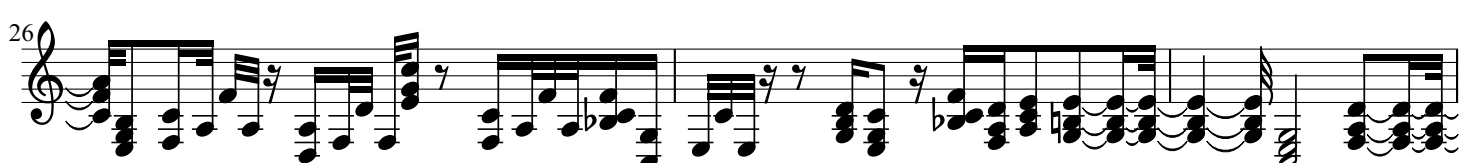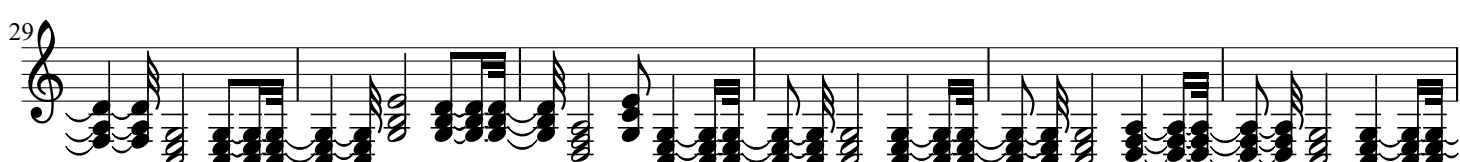

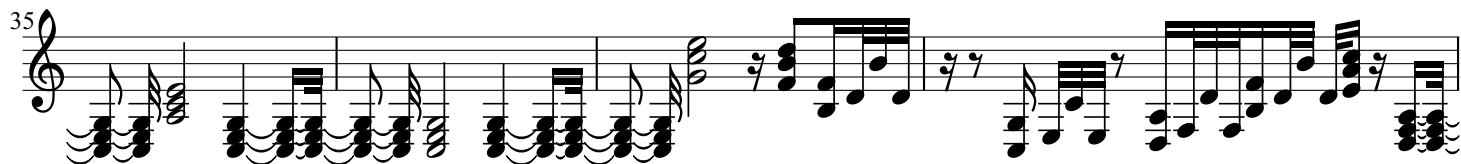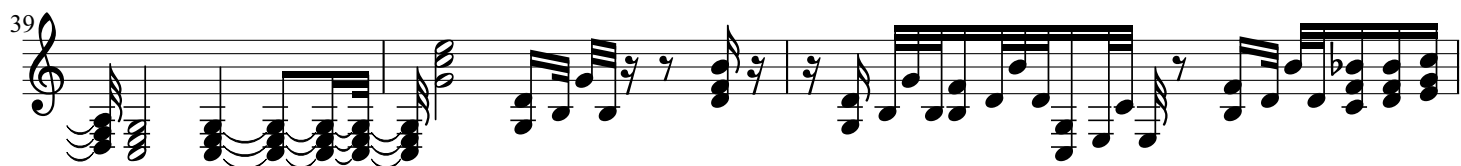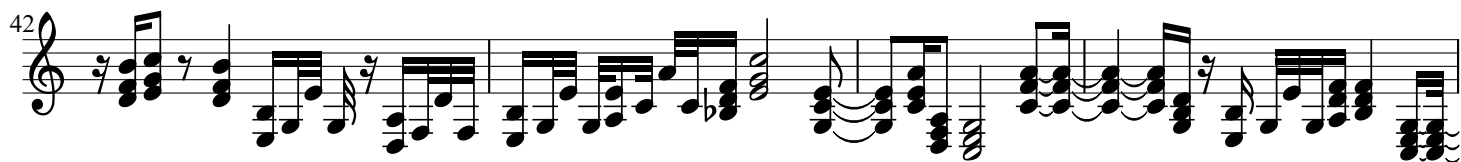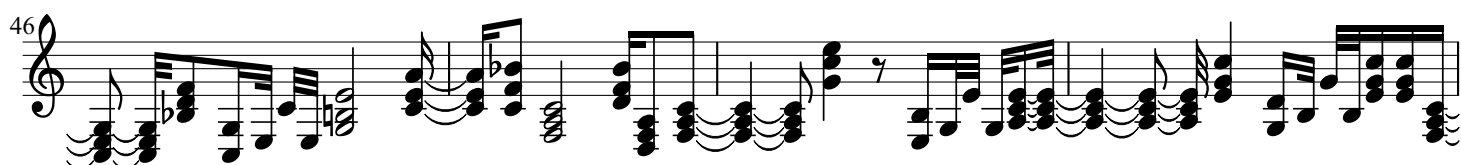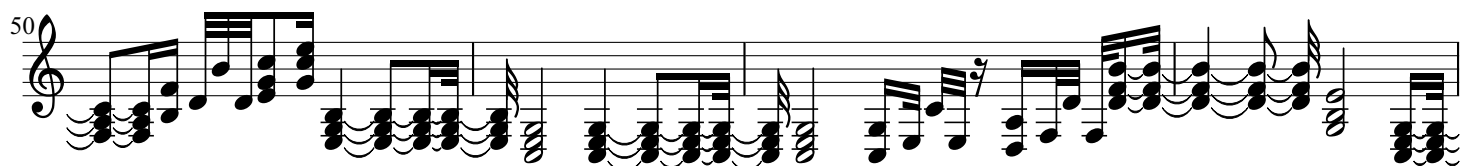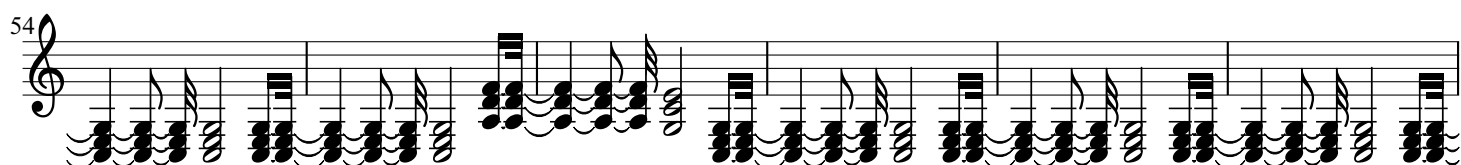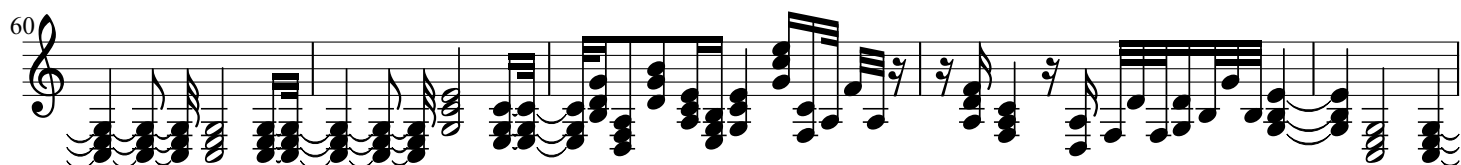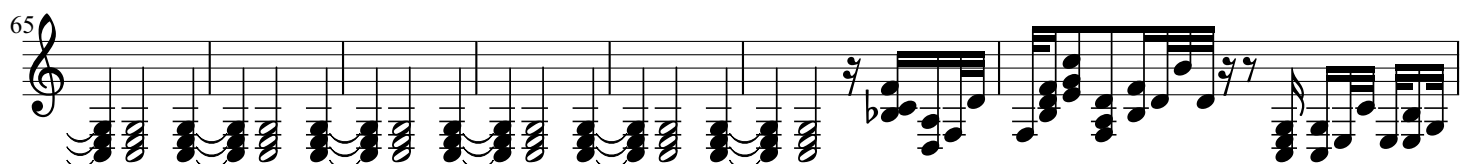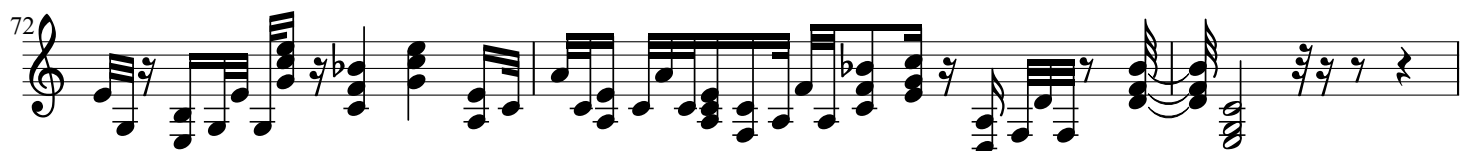

Supplement: Additional file 7: — Embryonic Stem Cell Long. (PDF 69 kb) [file 13148_2015_127_MOESM7_ESM.pdf]

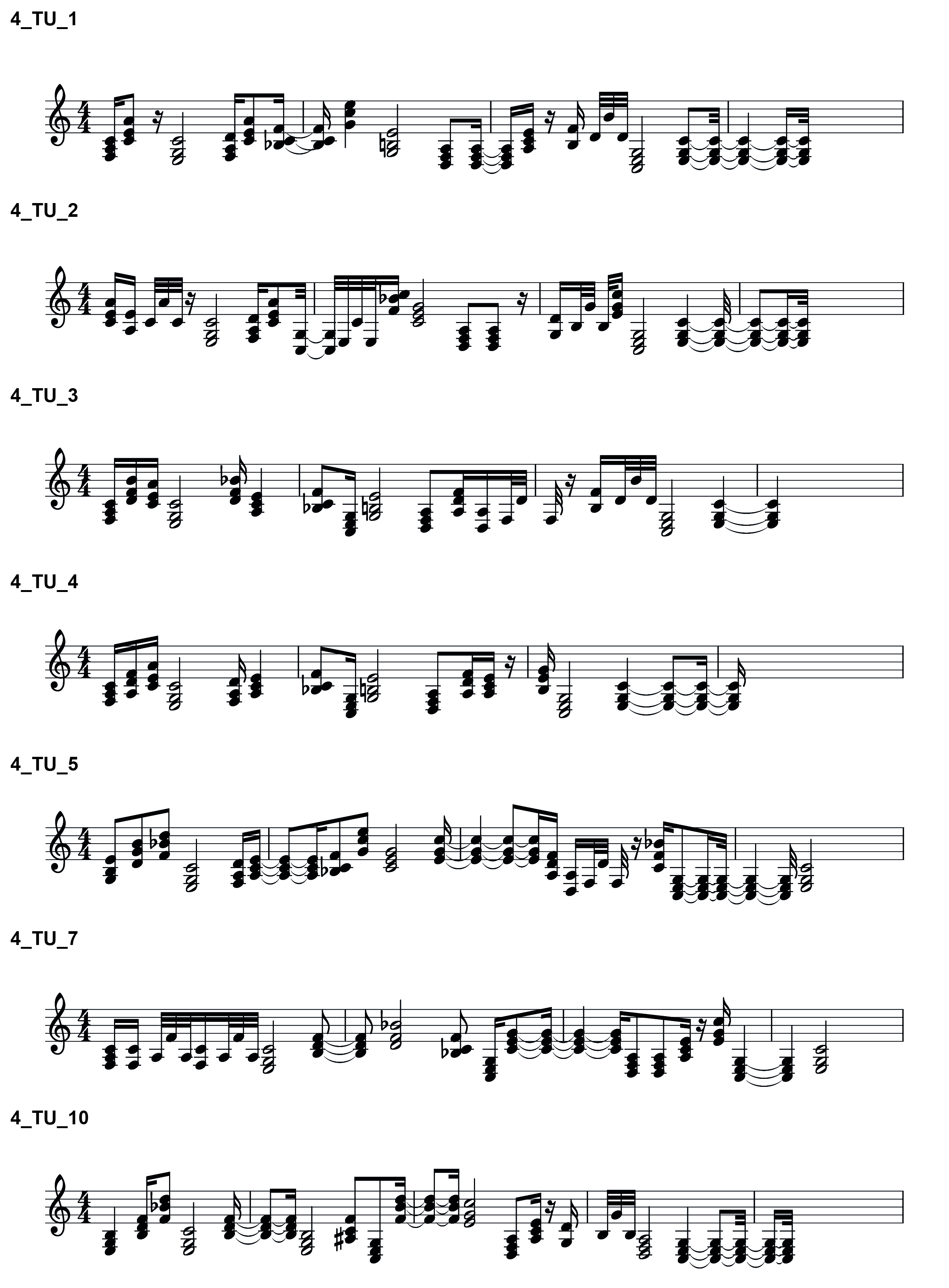

Supplement: Additional file 8: — Different Prostate Tumors. (TIFF 1,139 KB) [file 13148_2015_127_MOESM8_ESM.tiff]
